# Harm reduction associated with heated tobacco products: A systematic review and meta-analysis

**DOI:** 10.12669/pjms.41.1.10820

**Published:** 2025-01

**Authors:** Javed Akram, Shehla J. Akram, Nadia Naseem, Sheeraz Shehzad, Arslan Rana, Verda Ashraf, Ansa Akram, Usman E. Sheikh, Miland Joshi, Khalid S. Khan

**Affiliations:** 1 Professor Javed Akram, FRCP, Akram Medical Complex, Lahore, Pakistan; 2 Shehla J. Akram, PhD, Akram Medical Complex, Lahore, Pakistan; 3 Professor Nadia Naseem, PhD Department of Histopathology, University of Health Sciences Lahore, Pakistan; 4 Sheeraz Shehzad, MSPT Department of Physiotherapy, Ch Pervaiz Elahi Institute of Cardiology, Multan, Pakistan; 5 Arslan Rana, DPT Department of Physiotherapy, Laeeque Rafiq Institute of Health Sciences, Multan, Pakistan; 6 Verda Ashraf, FRCP Fatima Jinnah Medical University, Lahore, Pakistan; 7 Ansa Akram, DDS, Akram Medical Complex, Lahore, Pakistan; 8 Usman E. Sheikh, FRCP, Akram Medical Complex, Lahore, Pakistan; 9 Miland Joshi, PhD. Independent Chartered Statistician, Birmingham, UK; 10 Professor Khalid S. Khan, MSc. Department of Preventive Medicine and Public Health, University of Granada, Granada, Spain

**Keywords:** Systematic review, Meta-analysis, Harm reduction, Heated tobacco products, Smoking

## Abstract

**Objective:**

We conducted a systematic review and meta-analysis of randomised studies in humans comparing the outcomes of switching to heated tobacco products (HTPs) versus continuing conventional tobacco smoking by burning.

**Methods:**

We searched the electronic databases which included PubMed, Web of Science, Cochrane Controlled Trials Register, and Google Scholar from inception to May 2023. Randomised Controlled Trials (RCTs) in humans comparing HTPs with conventional burnt tobacco products were selected. Our search yielded 4817 search results, of which six RCTs (number of participants 1362, all from high-income countries) were selected. Five of the six included RCTs had low risk in four of five domains of bias; only one study had a high risk of bias in one domain. PRISMA guidelines were followed.

**Results:**

There were 40 biomarkers of harm reduction reported categorised into six categories. One cancer biomarker (nitrosamine) and two cardiovascular biomarkers (eosinophils and total bilirubin) showed statistically significant harm reduction (total NNAL SMD=0·82, 95% CI 0·67-0·98, eosinophils SMD=0·38, 95% CI 0·12-0·65, total bilirubin SMD=0·71, 95% CI 0·28-1·31). The remaining biomarkers of harm in cardiovascular, inflammatory, metabolic, pulmonary, and renal categories showed imprecise findings.

**Conclusions:**

In RCTs of moderate quality, some biomarkers show harm reduction associated with switching from burnt tobacco smoking to HTPs. The majority of the findings are imprecise due to the small sample sizes of the included studies. Sufficiently powered, robust RCTs targeting key harm reduction biomarkers within both upper and low-middle income country settings are required in the future.

## INTRODUCTION

Worldwide more than eight million people are killed each year by direct or indirect exposure to the harmful products of tobacco.^1^ It was reported by World Health Organization (WHO) in 2020 that 22.3% of the global population used tobacco, with 36.7% of men and 7.8% of women.^1^ Tobacco is the most commonly used through its combustion in the form of cigarettes, cigars, pipe tobacco, and *bidis*, etc.^1^ The dangers of inhaling toxicants from conventional or burnt tobacco products via cigarettes or cigars are well documented.^2^ Recently, new devices have been introduced into the market that heat tobacco instead of burning it.^3^ These work at relatively lower temperatures to produce an inhalable nicotine aerosol that may reduce toxicant exposure, when compared to the products that burn tobacco.^3^ These are distinct from e-cigarettes which use heat to activate an e-liquid that is primarily nicotine.^4^ These heat-not-burn (HNB) tobacco devices are made for conventional smokers utilizing burnt tobacco products who are aiming to quit. Harm reduction associated with the tobacco consumption is a key public health concern.^5^

In March 2022, the U.S Food and Drug Administration (FDA) granted one of the HNB tobacco device of a leading company as modified risk tobacco product (MRTP) on the basis of scientific evidence that switching completely from conventional cigarettes to the HNB tobacco system significantly reduces exposure to at least 15 harmful or potentially harmful carcinogens and toxic chemicals. FDA deemed marketing such products, not as safe or approved, but to be appropriate for the protection of public health. However rigorous post-marketing toxicity studies on consumers are still being carried out to review its authorization.^6^

Evidence synthesis can help in determining whether heated tobacco products (HTPs) lead to harm reduction. One previously published systematic review concluded that heated tobacco products lower the lifetime cancer risk for humans when compared to combustible tobacco products. However, other health effects were not evaluated.^7^ Another evidence synthesis that compared the biomarkers of potential harm in adult smokers switching from traditional burnt tobacco to e-cigarettes; however, it did not include a comparison with HTPs.^8^ A more recent meta-analysis documented significant reductions in biomarkers of exposure (BoE) in conventional cigarette smokers who switched to HTPs. However, this review did not shed light on whether the reduction in BoE was linked to harm reduction.^9^

A further systematic review also reported reductions in BoE among individuals who used e-cigarettes and HTPs when compared to conventional cigarettes.^10^ Yet another systematic review conducted with searches completed in January 2021 encountered limitations stemming from low quality and imprecision primarily due to the small sample sizes of the studies included.^11^ Since its publication, several new studies have emerged. For instance, when conducting a PubMed search in April 2023 specifically focussing on the data related to HTPs from the last two years, more than 280 new citations have appeared since January 2021. The new literature may improve the validity and reliability of meta-analyses in an updated systematic review.

There remains an uncertainty around whether HTPs are associated with harm reduction concerning health risks compared to continuing burnt tobacco consumption. Thus, we conducted an updated systematic review and meta-analysis of randomised controlled trials (RCTs) comparing the outcomes in human subjects who switched to HTPs versus those who continued using burnt tobacco products.

## METHODS

We conducted a systematic review following prospective registration (https://doi.org/10.17605/OSF.IO/D6MTX)^12^ at Open Science Framework Registries on December 25, 2022 and reported it in compliance with PRISMA guidelines.^13^

### Search strategy and selection criteria:

We conducted a systematic literature search in three electronic databases (PubMed/Medline, Web of Science, Cochrane Controlled Trials Register, and Google Scholar from inception until May 2023) to look for RCTs comparing outcomes of individuals who switched from conventional cigarettes to HTPs and those who continued to smoke conventional cigarettes. Observational studies were not considered due the known risk of bias in such designs. We used the following search strategy with Boolean operators and without language restriction with RCT filter applied: ((smoking OR cigarette smoking OR cigarette smokers OR burn tobacco OR cigarette) AND (electronic nicotine delivery systems OR tobacco use cessation devices OR heat tobacco OR heated tobacco OR tobacco heating OR tobacco heating devices OR tobacco heating system OR tobacco heating products OR tobacco vapor products OR carbon heated tobacco OR carbon heat OR heat not burn tobacco OR heat-not-burn tobacco OR heated tobacco products OR modified risk tobacco product OR mrtp OR htps OR iqos OR glo OR ploom OR fuse OR pulse OR teeps OR pax OR mok OR lil OR iuoc OR ths OR chtp OR thp OR htp OR ths2.2 OR thp1.0 OR eclipse OR revo OR ploomtach OR ismoke OR i-glo OR heatbar OR accord OR reynolds premier OR v2pro OR series 3 OR series 7)) AND (comparative study)).

We also searched reference lists of the selected RCTs and previously published systematic reviews^7^,^9^–^11^ for potential eligible studies. RCTs comparing outcomes between individuals using HTPs with no prior conventional cigarette exposure and conventional cigarette smokers were excluded. Animal or laboratory studies were also excluded. All search and selection procedures were carried out in duplicate (SS and RA). After removal of duplicates, the titles and abstracts were screened by two independent viewers in accordance with the inclusion criteria. In case of any conflict, it was resolved by arbitration. Full texts of selected RCTs were retrieved and reviewed by two independent reviewers for eligibility and a list of finally selected RCTs was created.

### Data extraction and quality assessment:

Data were extracted and quality was assessed in duplicate (SS and RA). We extracted the names of the authors, country, study period, duration, and inclusion and exclusion criteria along with the type of HTPs. Where there were missing numerical data required for meta-analysis, we contacted authors by email on two occasions, without responses received. We carefully assessed studies for outcomes that captured harm reduction, avoiding data extraction of BoE. There is no agreed listing of biomarkers with respect to their link to harm reduction *versus* exposure only. For example, nitrosamine has in some literature been regarded as BoE^14^ and in others it is regarded as a biomarker of harm.^8^ Judgement had to be applied in the choice of outcomes for data extraction concerning harm reduction. The quality of included studies was assessed using the Cochrane risk of bias tool (version 2) in accordance with its manual.^15^ There were five domains of bias including randomization process, deviations from intended intervention, missing outcome data, outcome measurement, and selection of reported results.

### Data synthesis:

For each harm reduction biomarker, meta-analysis was carried out using the R package metagen, with random effects models where there was evidence of heterogeneity of study participants and fixed (or common) effects otherwise. Forest plots were prepared. The meta-analysis used standardised mean differences (SMDs) to allow for different units of measurements. If the reported estimate of effect is RE then SMD = RE/s where s is the pooled standard deviation of the effect, pooled across the comparison arms.

The pooled standard deviation s is estimated by SE/r where SE is the standard error of RE, and calculated by dividing the length of the CI for RE (as reported in the selected papers) by 3·92, and r is √[(1/m) + (1/n)] where m and n are the respective sizes of the two comparison arms. SE was estimated using the lower and upper confidence limits for the effect L and U by (U-L)/3·92, on the assumption of symmetric CIs with each limit of 1·96 SEs from the estimate. The value of s was then estimated from the SE as indicated previously. This gave the value of the SMD. For the SE of the SMD we used the formula: SE_SMD_ = √[(n_1_+n_2_)/n_1_n_2_))+(SMD^2^/2(n_1_+n_2)_)] from Harrer et al.^16^

In evaluating the results from Ludicke et al^17^, presented in its Table-II, we did not consider that the one-sided p values were suitable for our analysis, given that one-sided significance levels are hard to justify. We therefore converted the one-sided p-value levels to two-sided ones (by doubling), used these as attained significance levels to estimate the corresponding SE, and then adjusted the CIs so that they would be 95% CIs, on the simplifying assumption of symmetry and that the limits would be 1·96 standard errors from the estimate. Our estimated CIs are therefore slightly different from the ones in Lüdicke, though not greatly, and should not make any substantial differences to the findings.^16^ Where changes in biomarkers were reported as % ratios we transformed them so that the null value of no difference was zero, by the rule x -> ln (x/100) (ln is the natural log, to the base e). This needed to happen with biomarkers reported in Table-II in Haziza et al^18^ and Table-III in Ludicke et al.^19^

**Table-I:** Summary characteristics of participants of included studies in systematic review of harm reduction associated with heated tobacco products.

Author, Year	Country	Setting	Study years	Study period (Months)	Sample size in reported results	Mean age	BMI	Funding
Roethig HJ et al. 2008	USA*	Two clinical study centers	··	··	92 (M=45, F=52)	42.1	25.7	Philip Morris USA
Leroy CM et al. 2012	Poland	Single center	2007-2008	Oct-2007 to April-2008 (7)	309 (M=161, F=155)	43.6	24.84	Philip Morris International
Lüdicke F et al. 2017	Japan	Single center	··	··	117	··	··	Philip Morris Products S. A
Haziza C et al. 2019	USA	Four sites	2013-2014	Dec-2013 to Oct-2014 (10)	79 (M=72, F=49)	36.45	26.4	Philip Morris International
Lüdicke F et al. 2019	USA	20 study sites	2015-2016	March 2015 - September 2016	673 (M=397, F=276)	44.7	27	Philip Morris International
Bosilkovska M et al. 2020	Poland	Single center	2016	Jan-Aug (8)	92 (M=64, F=56)	38.9	25.73	Philip Morris International

* Country of corresponding author.

**Table-II:** Risk of bias of included studies in systematic review of harm reduction associated with heated tobacco products.

Author, Year	Country	Randomization process	Deviation from intended intervention	Missing outcome data	Measurement of outcome	Selection of the reported results	Overall result
Roethig HJ et al. 2008	USA*	Low	Some concerns	Low	Low	Low	Some concerns
Leroy CM et al. 2012	Poland	Low	Some concerns	Low	Low	Low	Some concerns
Lüdicke F et al. 2017	Japan	Low	Some concerns	Low	Low	Low	Some concerns
Haziza C et al. 2019	USA	Low	Some concerns	High	Low	Low	High
Lüdicke F et al. 2019	USA	Low	Some concerns	Low	Low	Low	Some concerns
Bosilkovska M et al. 2020	Poland	Low	Some concerns	Low	Low	Low	Some concerns

* Country of corresponding author.

**Table-III:** Results of harm reduction associated with heated tobacco products.

Name of biomarker	Category of harm reduction	Number of studies with biomarker	Number of studies with meta-analyzable data	Summary result (SMD)	95% confidence interval
Total NNAL (4-(methylnitrosamino)-1-(3-pyridyl)-1-butanol)	Cancer	1	1	0.75*	0.59; 0.91
8-epi-prostaglandin F2α	Cardiovascular	6	5	1.47	-0.83; 3.77
11-dehydrothromboxane B2	Cardiovascular	6	4	0.06	-0.56; 0.68
Fibrinogen	Cardiovascular	5	4	0.03	-0.45; 0.52
Hs-CRP (High sensitivity-C-reactive protein)	Cardiovascular	4	3	0.73	-0.64; 2.09
Homocysteine	Cardiovascular	4	4	2.39	-2.30; 7.08
s-ICAM-1 (soluble intercellular adhesion molecule-1)	Cardiovascular	4	3	-0.14	-0.38; 0.10
Diastolic blood pressure	Cardiovascular	3	3	-0.02	-0.26; 0.22
Systolic blood pressure	Cardiovascular	3	3	-0.04	-0.39; 0.46
Myeloperoxidase	Cardiovascular	2	2	-0.00	-1.69; 1.68
Platelet count	Cardiovascular	2	2	-0.03	-0.25; 0.19
von Willebrand factor	Cardiovascular	2	1	-0.18	-0.44; 0.08
8-OHdG (8-Hydroxy-2-deoxyguanosine)	Cardiovascular	1	1	0.00	-0.42; 0.42
ADP (adenosine diphosphate)-induced platelet aggregation amplitude	Cardiovascular	1	1	0.08	-0.18; 0.34
ADP (adenosine diphosphate)-induced platelet aggregation slope	Cardiovascular	1	1	0.01	-0.25; 0.27
Basophils	Cardiovascular	1	1	0.07	-0.19; 0.33
Eosinophils	Cardiovascular	1	1	0.38*	0.12; 0.64
Hematocrit	Cardiovascular	1	1	0.22	-0.04; 0.48
Lymphocytes	Cardiovascular	1	1	-0.02	-0.28; 0.24
Monocytes	Cardiovascular	1	1	0.08	-0.18; 0.34
Neutrophils	Cardiovascular	1	1	-0.01	-0.27; 0.25
Ox LDL (Oxidized low-density lipoprotein)	Cardiovascular	1	1	-0.02	-0.28; 0.24
RBC (Red blood cell) count	Cardiovascular	1	1	0.15	-0.11; 0.41
Total antioxidant capacity	Cardiovascular	1	1	0.00	-0.42; 0.42
Total Bilirubin	Cardiovascular	1	1	0.71*	0.28; 1.31
White blood cell (WBC) count	Inflammatory	4	4	-0.28	-0.66; 0.10
IL-6 (Interleukin-6)	Inflammatory	1	1	-0.08	-0.34; 0.18
HDL cholesterol (High-density lipoprotein)	Metabolic	6	5	0.18	-0.09; 0.45
LDL cholesterol (Low-density lipoprotein)	Metabolic	5	4	-0.07	-0.25; 0.11
Total cholesterol	Metabolic	4	4	-0.67	-2.88; 1.54
Triglycerides	Metabolic	4	3	-0.06	-0.30; 0.18
Glucose	Metabolic	3	3	-0.16	-1.05; 0.74
Weight	Metabolic	3	3	0.00	-0.24; 0.24
Waist circumference	Metabolic	3	3	0.09	-0.14; 0.33
HbA1c (Hemoglobin A1c)	Metabolic	3	3	-0.00	-0.24; 0.24
Apolipoprotein A1	Metabolic	2	2	0.05	-0.26; 0.35
Apolipoprotein B	Metabolic	2	2	-0.17	-0.48; 0.13
Hemoglobin	Metabolic	1	1	0.17	-0.09; 0.43
FEV1 (Forced expiratory volume in 1 second)	Pulmonary	4	4	0.16	-0.08; 0.40
Urine microalbumin	Renal	1	1	-0.08	-0.50; 0.35

* Statistically significant; SMD=Standardized Mean Difference, see method section for detail; see Appendix-3 for related forest plots; A positive SMD value indicates higher biomarker level in smokers versus those who switched to heat not burn devices.

### Role of funding source:

There was no funding for the work. It is an investigator-initiated project.

## RESULTS

**Fig.1:**
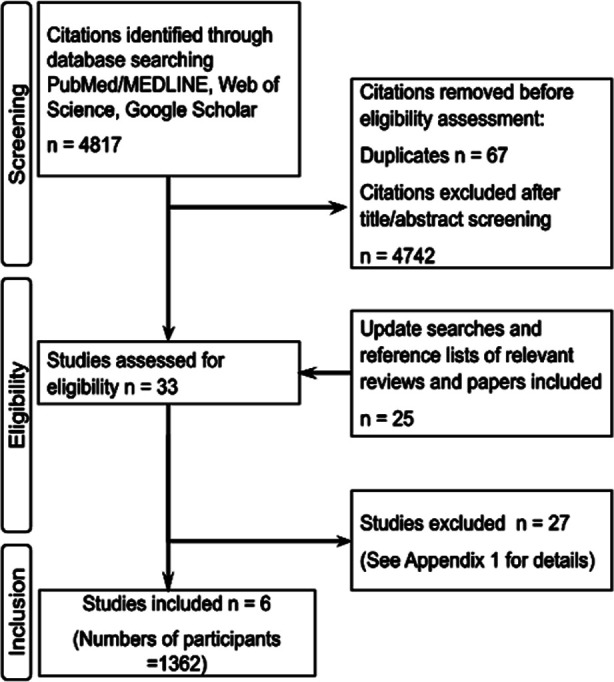
Flow diagram of study selection in systematic review of harm reduction associated with heated tobacco products.

Our initial search yielded 4817 citations. After removal of duplicates (n=67), 4750 citations were screened for eligibility on the basis of titles and abstracts (Fig.1). Full papers were evaluated for 33 citations. After full text assessment, six RCTs (number of participants 1362) were finally selected. All 27 excluded studies with reasons for rejection are listed in Appendix-1. The major reason for exclusion of full texts (n=20) was the lack of information related to harm reduction biomarkers; other reasons were non-randomised design, studies without HTPs, non-human studies, and no comparison of continuing burn tobacco smoking *versus* switching to HTPs.

### Study characteristics and quality:

All included RCTs in our systematic review and meta-analysis were from high-income countries namely the United States of America (USA), Poland and Japan. There were at least two comparison groups present in each included RCT; one group had conventional cigarette smokers who continued to smoke conventional cigarettes and the other group had conventional cigarette smokers who switched to HTPs. The participants who switched to HTPs used different devices having the same underlying mechanism of heating tobacco. The devices were: second generation electrically heated cigarette smoking system (EHCSS),^14^ EHCSS series-K cigarette (EHCSS-K6),^20^ menthol Tobacco Heating System 2.2 (mTHS),^18^,^19^ Tobacco Heating System 2.2 (THS) IQOS,^17^ and Carbon-Heated Tobacco Product 1.2 (CHTP).^21^ The summary characteristics of included RCTs are provided in Table-I. The details of participants’ inclusion and exclusion criteria, and descriptions of comparison groups are presented in Appendix-2. The summary of the quality of included RCTs is presented in Table-II. Five^14^,^17^,^19^–^21^ of the total six RCTs had a low risk of bias across four domains whereas one RCT^18^ suffered from a high risk of bias in one domain of missing outcome data. The details of the risk of bias assessment are provided in Appendix-3.

### Data synthesis:

There were 40 biomarkers in six harm reduction categories reported as shown in Table-III. All forest plots are provided in Appendix-4. One cancer biomarker (nitrosamine) and two cardiovascular biomarkers (eosinophils and total bilirubin) showed statistically significant harm reduction (total NNAL SMD=0·82, 95% CI 0·67-0·98, eosinophils SMD=0·38, 95% CI 0·12-0·65, total bilirubin SMD=0·71, 95% CI 0·28-1·31). The findings of the remaining 37 biomarkers were insignificant. Individual RCTs showed changes in harm biomarkers but meta-analysis showed imprecise findings.

*(Information contained in Appendix I to IV is available on request)*

## DISCUSSION

This systematic review presented comparative information related to changes in harm reduction biomarkers among conventional cigarette smokers who switched to HTPs. Among RCTs with a moderate level of quality conducted in high-income country settings, some biomarkers demonstrated harm reduction while the others yielded imprecise findings. In particular, one cancer and two cardiovascular related biomarkers demonstrated statistically significant association with switching to HTPs.

Our review was reported in line with the PRISMA checklist.^13^ To our knowledge, this is the first systematic review that has focused entirely on harm reduction associated with switching from conventional smoking to HTPs among human subjects. Our search was comprehensive; hence we are confident that all the relevant published studies have been captured. However, the number of RCTs included was too small to assess for the funnel plot asymmetry that helps identify potential publication and related biases. The quality of the studies included depict both a risk of bias and imprecision in their findings. Not all harm reduction biomarkers were reported in all included RCTs. In our forest plots, we demonstrated explicitly where biomarker data were missing (Appendix-4). Thus, most of the meta-analyses conducted lacked statistical power and 95% CIs were wide including the possibility of no association. As there were multiple measures of outcomes, including a mixture of reporting change-from-baseline or final value scores, we deployed SMD to unify biomarker data for meta-analysis. Some outcome data required statistical manipulation to generate meta-analysable data as detailed in the methods. Considering the heterogeneity arising from variations among the participants, devices, and biomarkers, we used a random effects model which provides more conservative estimates. In summary, ours is the most up-to-date and robust meta-analysis that synthesizes the evidence on harm reduction associated with HTPs.

There is a lack of consensus in the literature concerning biomarkers of harm reduction. For instance, while we considered carboxyhemoglobin as a BoE, occasional literature has categorized it as a biomarker of harm.^17^ This is an important gap limiting the ability to design and conduct future research focussing on outcomes that are most relevant for public health. Another significant gap is the scarcity or absence of studies from low-middle-income countries. These issues ought to be addressed as a priority in moving forward. The interpretation of our findings should be made in light of previously published literature. A recent systematic review revealed that the lifetime cancer risk in human subjects was lower among users of HTPs compared to those who smoked traditional combustible tobacco products.^7^

Our meta-analysis showed comparable findings. Statistically significant reductions in BoE in cigarette smokers who switched to HTPs were reported in another previously published systematic review.^9^ We evaluated only harm reduction biomarkers and found significant changes in some biomarkers in HTPs users. In the Copenhagen general population study conducted in 2020 that recruited more than one hundred thousand smokers, both observational and genetic analysis revealed a causal link between higher daily tobacco consumption and reduced plasma bilirubin levels. The authors concluded that bilirubin might be a triggering factor of smoking-induced respiratory diseases.^22^ They further concluded that bilirubin might be a triggering factor of smoking-induced respiratory diseases.

Our review has also given an insight into the potential harm reduction associated with switching from the burnt tobacco products (cigarettes, cigars, *bidis* etc) to HTPs. However, it’s worth noting that the findings from the included RCTs did not yield conclusive results. Within tobacco control policies worldwide, harm reduction associated with HTPs is a key element of public health strategy. A recently published study has reported that more than one third of the participants discontinued smoking cigarettes by switching to HTPs.^23^ Alternatively, nicotine delivery devices such as e-cigarettes have also shown the similar results.^23^ These substitutes of conventional cigarette smoking are evolving as an important component of harm reduction strategies throughout the world. In this regard, our meta-analysis implies that HTPs are less harmful than the conventional cigarettes and cigars, but there remains a need for further research to establish their potential role.

## CONCLUSION

Total avoidance of smoking is the best advice for health. Initiation of HTPs is not recommended for non-smokers. The ideal remedy for smokers is to quit tobacco all together. In human RCTs of moderate quality among smokers unable to quit and switching to HTPs within high-income country settings some biomarkers demonstrated harm reduction while the rest demonstrated imprecise findings due to small sample sizes. Large, robust trials in both upper and low-middle income country settings are required to assess the potential of harm reduction with various HTPs among those not planning to quit smoking.

### Authors’ Contribution:

**JA and KSK**: The idea of this systematic review was conceived.

**SS and RA** did the data curation.

**MJ**: Visualisation and formal analysis were carrie.

**KSK, RA, and SS:** This was an investigator-initiated project. Investigation regarding this topic was done.

**KSK, SS and RA**: Methodology was constructed by KSK and MJ. The original draft was written.

**KSK, JA, UES, NN, AA, AV, and SJA:** Review and editing was done.

**KSK:** The overall supervision was provided.

All authors had full access to all the data in the study and had final responsibility for the decision to submit for publication.

## References

[1] Tobacco https://www.who.int/news-room/fact-sheets/detail/tobacco.

[2] Prevention (US) C for DC and, Promotion (US) NC for CDP and H, Health (US) O on S and (2010). Chemistry and Toxicology of Cigarette Smoke and Biomarkers of Exposure and Harm. Centers for Disease Control and Prevention (US).

[3] Evaluation of the Tobacco Heating System 2.2 Part 2:Chemical composition, genotoxicity, cytotoxicity, and physical properties of the aerosol - ScienceDirect.

[4] Health CO on S and Smoking and Tobacco Use;Electronic Cigarettes (2022). Centers for Disease Control and Prevention. Published November 18.

[5] Yach D (2022). Tobacco harm reduction matters. Lancet.

[6] Products C for T. Philip Morris Products S. A. Modified Risk Tobacco Product (MRTP) Applications. FDA https://www.fda.gov/tobacco-products/advertising-and-promotion/philip-morrisproducts-sa-modified-risk-tobacco-product-mrtp-applications.

[7] Kusonić D, Bijelić K, Kladar N, Božin B, Torović L, SrđenovićČonić B (2023). Comparative Health Risk Assessment of Heated Tobacco Products versus Conventional Cigarettes. Subst Use Misuse.

[8] Hartmann-Boyce J, Butler AR, Theodoulou A, Onakpoya IJ, Hajek P, Bullen C (2023). Biomarkers of potential harm in people switching from smoking tobacco to exclusive e-cigarette use, dual use or abstinence:secondary analysis of Cochrane systematic review of trials of e-cigarettes for smoking cessation. Addiction.

[9] Human Biomarker Exposure from Cigarettes Versus Novel Heat-Not-Burn Devices:A Systematic Review and Meta-Analysis |Nicotine &Tobacco Research |Oxford Academic https://academic.oup.com/ntr/article-abstract/22/7/1077/5602686?redirectedFrom=fulltext&login=false.

[10] Akiyama Y, Sherwood N (2021). Systematic review of biomarker findings from clinical studies of electronic cigarettes and heated tobacco products. Toxicol Rep.

[11] Tattan-Birch H, Hartmann-Boyce J, Kock L, Simonavicius E, Brose L, Jackson S (2022). Heated tobacco products for smoking cessation and reducing smoking prevalence. Cochrane Database Syst Rev.

[12] Akram PJ, Shehzad S, Khan PK, Akram DSJ, Rana A Harm reduction associated with heat-not-burn tobacco products:A systematic review and meta-analysis of randomised trials. https://osf.io/jxfm2/.

[13] Rethlefsen ML, Kirtley S, Waffenschmidt S, Ayala AP, Moher D, Page MJ, PRISMA-S Group (2021). PRISMA-S:an extension to the PRISMA Statement for Reporting Literature Searches in Systematic Reviews. Syst Rev.

[14] Roethig HJ, Feng S, Liang Q, Liu J, Rees WA, Zedler BK (2008). A 12-Month, Randomized, Controlled Study to Evaluate Exposure and Cardiovascular Risk Factors in Adult Smokers Switching from Conventional Cigarettes to a Second-Generation Electrically Heated Cigarette Smoking System. J Clin Pharmacol.

[15] Higgins JPT, Altman DG, Gøtzsche PC, Jüni P, Moher D, Oxman AD (2011). The Cochrane Collaboration's tool for assessing risk of bias in randomised trials. BMJ.

[16] Harrer M, Pim Cuijpers, Toshi Furukawa, Ebert D Doing Meta-Analysis with R:A Hands-On Guide (1st ed.). Chapman and Hall/CRC.

[17] Lüdicke F, Ansari SM, Lama N, Blanc N, Bosilkovska M, Donelli A (2019). Effects of Switching to a Heat-Not-Burn Tobacco Product on Biologically Relevant Biomarkers to Assess a Candidate Modified Risk Tobacco Product:A Randomized Trial. Cancer Epidemiol Biomarkers Prev.

[18] Haziza C, De La Bourdonnaye G, Donelli A, Skiada D, Poux V, Weitkunat R (2020). Favorable Changes in Biomarkers of Potential Harm to Reduce the Adverse Health Effects of Smoking in Smokers Switching to the Menthol Tobacco Heating System 2.2 for 3 Months (Part 2). Nicotine Tob Res.

[19] Lüdicke F, Picavet P, Baker G, Haziza C, Poux V, Lama N (2018). Effects of Switching to the Menthol Tobacco Heating System 2.2, Smoking Abstinence, or Continued Cigarette Smoking on Clinically Relevant Risk Markers:A Randomized, Controlled, Open-Label, Multicenter Study in Sequential Confinement and Ambulatory Settings (Part 2). Nicotine Tob Res.

[20] Leroy MC, Dziedzic JK, Ancerewicz J, Lindner D, Kulesza A, Magnette J (2012). Reduced exposure evaluation of an Electrically Heated Cigarette Smoking System. Part 7: A one-month, randomized, ambulatory, controlled clinical study in Poland. Regul Toxicol Pharmacol.

[21] Bosilkovska M, Tran CT, De La Bourdonnaye G, Taranu B, Benzimra M, Haziza C (2020). Exposure to harmful and potentially harmful constituents decreased in smokers switching to Carbon-Heated Tobacco Product. Toxicol Lett.

[22] Kodal JB, Çolak Y, Kobylecki CJ, Vedel-Krogh S, Nordestgaard BG, Afzal S (2020). Smoking Reduces Plasma Bilirubin:Observational and Genetic Analyses in the Copenhagen General Population Study. Nicotine Tob Res Off J Soc Res Nicotine Tob.

[23] Caponnetto P, Campagna D, Maglia M, Benfatto F, Emma R, Caruso M (2023). Comparing the Effectiveness, Tolerability, and Acceptability of Heated Tobacco Products and Refillable Electronic Cigarettes for Cigarette Substitution (CEASEFIRE):Randomized Controlled Trial. JMIR Public Health Surveill.

